# The *in vitro* assessment of enamel remineralization and cytotoxicity of experimental silver diamine fluoride

**DOI:** 10.2340/biid.v13.46612

**Published:** 2026-08-05

**Authors:** Pailin Tribuppachatsakul, Piyaphong Panpisut, Dhananthat Chawhuaveang, Ollie Yiru Yu, Somying Patntirapong, Piyaporn Pultanasarn

**Affiliations:** a Faculty of Dentistry, Thammasat University, Pathum Thani, Thailand; b Faculty of Dentistry, The University of Hong Kong, Hong Kong, China

**Keywords:** Silver diamine fluoride, silver nitrate, enamel, remineralization, sodium fluoride

## Abstract

**Objective:**

This in vitro study evaluated the enamel remineralization potential and cytotoxicity of an experimental 38% silver diamine fluoride prepared using a silver nitrate precursor (EXP-SDF; Dentalife, Melbourne, Australia). EXP-SDF was compared with commercial 38% SDF (Topamine; Dentalife), 5% NaF varnish (Duraphat; Colgate-Palmolive, USA), and 2.1% NaF gel (ClinproClear; 3M ESPE Dental Products, USA).

**Materials and methods:**

Fluoride and silver concentrations, as well as the pH of EXP-SDF, were initially examined. Enamel specimens were prepared and randomized into five groups: Topamine, EXP-SDF, Duraphat, ClinproClear, and deionized water (control). The enamel specimens were demineralized and treated with materials and then subjected to a 14-day pH-cycling regimen. Enamel surface remineralization was quantified as the percentage of surface microhardness recovery (%SMHR). Mineral precipitation on the surface was then examined using Raman spectroscopy, X-ray diffraction (XRD), and scanning electron microscopy with energy-dispersive X-ray spectroscopy (SEM-EDX). Cytotoxicity of the experimental SDF was also evaluated using the 3-(4,5-dimethylthiazol-2-yl)-2,5-diphenyltetrazolium bromide (MTT) assay.

**Results:**

EXP-SDF exhibited a higher fluoride concentration but comparable pH to Topamine. The increase in enamel surface microhardness of EXP-SDF (35%) was similar to that of Topamine (34%) (*p* = 0.98). The SDF groups also showed significantly higher %SMHR than Duraphat (−7%) and ClinproClear (−45%) (*p* < 0.05). Raman spectroscopy showed a reduction in the phosphate peak after demineralization, but the peaks after pH cycling were comparable across all groups. XRD patterns identified hydroxyapatite peaks in all groups, with the silver-containing phases only detected in the SDF-treated groups. SEM-EDX revealed high levels of Ag and F on the enamel surface of both Topamine and EXP-SDF. The cell viability of Topamine (77%) was also comparable to that of EXP-SDF (70%) (*p* = 0.32).

**Conclusion:**

EXP-SDF promoted comparable remineralization of demineralized enamel and *in vitro* cytotoxicity comparable to that of commercial SDF. The remineralizing effects of SDF groups were also higher than those of professional NaF products.

**Key messages**

- The experimental 38% silver diamine fluoride (SDF) prepared using a nitrate precursor exhibited enamel remineralizing action and cytotoxicity comparable to those of commercial 38% SDF.
- The experimental and commercial SDF groups demonstrated superior enamel remineralization compared with NaF products (5% NaF varnish and 2.1% NaF gel).

## Introduction

Caries is the most prevalent noncommunicable disease worldwide, posing a major health, social, and economic burden [1]. Caries is a dynamic disease associated with the imbalance between demineralization and remineralization; when left untreated, it can lead to severe pain and infection [2]. Hence, modulating the imbalance by enhancing remineralization as early as possible, when the lesion is still in the noncavitation stage, is considered an effective management strategy [3].

The management of early enamel caries lesions is typically recommended using 5% sodium fluoride varnish (NaF, ~22,000 ppmF) applied every 3–6 months, which has been shown to achieve an approximately 64% caries-arrest rate [4, 5]. However, NaF varnish requires frequent applications, and its effectiveness varies with patient risk and adherence to recall appointments [6]. Silver diamine fluoride (38% SDF; ~50,000 ppmF) may be more effective at arresting carious lesions due to its superior remineralizing and antibacterial effects [7, 8]. A decision-analytic model in preschoolers reported that SDF outperformed NaF for managing cavitated lesions, resulting in lower costs and greater caries control [9]. The permanent black staining of treated lesions is a significant barrier to patient and parental acceptance of SDF, raising esthetic concerns and increasing the risk of negative social judgment [10, 11].

While SDF is typically recommended for active cavitated lesions, a clinical study reported superior caries arrest in enamel caries with 38% SDF compared with 5% NaF varnish at 6 months [12]. A split-mouth clinical trial also reported a higher rate of enamel caries arrest with 38% SDF (74.4%) than with 5% NaF varnish (67.5%), though the difference was not statistically significant [13]. *In vitro* studies further support the superior remineralizing performance of SDF, demonstrating shallower lesion depths, greater mineral density, and higher gains in enamel microhardness [14, 15].

In general, 38% SDF is formulated from silver fluoride, ammonium fluoride, ammonium solution, a colorant, and water. A manufacturer proposed that substituting the silver source with silver nitrate (in combination with hydrofluoric acid) could yield a 38% SDF product at a lower per-unit cost [16]. The previous study demonstrated similar dentin remineralization actions for SDF prepared with a silver nitrate precursor (Dentalife, Melbourne, Australia) compared with the original formulation [16]. Additionally, the manufacturer reported lower black staining with the new formulation. By addressing both esthetic concerns and affordability, such a formulation may facilitate wider adoption of SDF in visible enamel surfaces. In addition, lower manufacturing costs may facilitate the commercial rationale for pursuing expanded regulatory approval for caries treatment. Evidence for enamel remineralization with the modified SDF formulation prepared using a silver nitrate precursor remains limited.

The objective of the current study was therefore to characterize and investigate the enamel remineralization of the experimental SDF prepared with a nitrate precursor (EXP-SDF) compared with the commercial SDF and NaF products. The null hypothesis was that the experimental SDF would exhibit no significant difference in the remineralization and cytotoxic effects compared with the commercial materials.

## Materials and methods

### Materials characterization

The materials used in the present study are provided in Table 1. The precursors used for the preparation of the commercial SDF formulation (Topamine) and experimental SDF (EXP-SDF) are provided in Table 1. However, the exact composition of the experimental SDF product was not disclosed by the manufacturer.

**Table 1:** The topical fluoride used in the current study.

Materials (Abbreviation)	Composition	Suppliers	Lot number
Commercial 38% SDF (Topamine)	Precursor: Silver fluoride, ammonium solution, brilliant blue solution, process water Product: 36–40% Silver diamine fluoride, 24–27% soluble silver ions, with < 0.02% other components, dissolved in water to 100%	Dentalife, Ringwood, Victoria, Australia	B1552.1
Experimental 38% SDF (EXP-SDF)	Precursor: Silver nitrate, hydrofluoric acid, ammonia gas, brilliant blue solution, process water Product: 36–40% Silver diamine fluoride, 24–27% soluble silver ions, with < 0.02% other components, dissolved in water to 100%	Dentalife, Ringwood, Victoria, Australia	DF250124C
5% NaF varnish (Duraphat)	Ethanol (30–40 wt%), propylene glycol (5–10 wt%), sodium fluoride (5–10 wt%), menthol (3–5 wt%), L-menthol (1–3 wt%), D-limonene (1–3 wt%), vanillin (1–3 wt%), trans-menthone (0.1–1 wt%)	Colgate-Palmolive Co., New York, NY, USA	268199
2.1% NaF gel (ClinproClear)	Sweetener (< 5wt%), phosphate salt (< 3 wt%), sodium fluoride (< 3 wt%), ethyl alcohol (< 2wt%), buffer (< 1wt%)	3M ESPE Dental Products, St. Paul, MN, USA	116811297

The actual amount, compositions, and precursors used for SDF preparation were not provided by the manufacturer. SDF: silver diamine fluoride.

The concentrations of Ag and the pH level of SDF were analyzed descriptively. Silver ion concentration in Topamine and EXP-SDF was analyzed (*n* = 1) using inductively coupled plasma optical emission spectrometry (ICP‑OES; Optima 8300, PerkinElmer, Shelton, CT, USA). Measurements were acquired at 328.068 nm over a working range of 0.1–50 mg/L. Calibration was performed using an environmental standard containing 26 components (CPA Chem, Bulgaria). The pH of Topamine and EXP-SDF was additionally measured using a pH meter (Orion Star, Thermo Scientific, Waltham, MA, USA) in five replicate measurements per material. The instrument was calibrated at 25 ± 1 °C using standard buffer solutions (pH 4.0, 7.0, and 10.0).

Fluoride release from Topamine, EXP-SDF, Duraphat, and ClinproClear was measured using the fluoride-specific electrode (Orion 9609BNWP, Thermo Fisher Scientific, Waltham, MA, USA). The calibration was performed at 0.1, 1, 10, 100, and 1000 ppm. Topamine and EXP-SDF were diluted by adding 10 µL of the material to 9990 µL of deionized water (final dilution 1:1000, v/v). For Duraphat and ClinproClear, 0.01 g of material was dissolved in 10 mL of deionized water. Then, the dilution was mixed with TISAB II at a 1:1 (v/v) ratio prior to measurement with the electrode. The measurement was performed for five replications.

### Surface microhardness recovery of enamel

**Figure 1:**
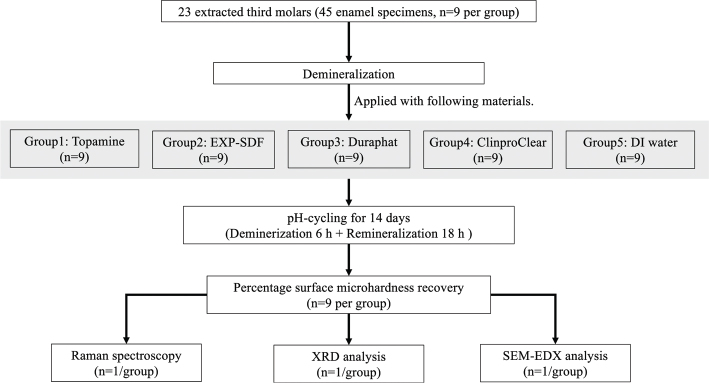
The experimental flowchart of the current study.

The percentage of surface microhardness recovery (%SMHR) of demineralized enamel was the main test to compare the *in vitro* remineralizing effects of each material in the current study (Figure 1). The sample size for enamel specimens in the study was estimated using G*Power software (version 3.1.9.7) [17] for a one-way analysis of variance (ANOVA) with five experimental groups, based on the effect size calculated from the previous study [18]. The test indicated that *n* = 9 per group (total of 45 specimens) was required to achieve power greater than 0.95 at an alpha level of 0.05.

The collection of 23 extracted human third molars (two enamel specimens per tooth) was approved by the Human Research Ethics Committee of Thammasat University (Science) (HREC‑TUSc, COE No. 009/2568; Project No. 68DE031). The requirement for written informed consent was waived because no patient-identifiable information was obtained. The extracted teeth were kept in 0.1% thymol solution at room temperature until use. Teeth with visible cracks, caries, or enamel defects identified under a stereomicroscope were excluded. All teeth were cleaned to remove residual soft tissue and surface contaminants.

Each tooth was sectioned vertically to obtain two enamel specimens from the buccal and lingual surfaces. The enamel surfaces were sequentially ground using silicon carbide abrasive papers with grit sizes of 1200, 2400, and 4000 under water cooling. Specimens were examined under a stereomicroscope, and those exhibiting cracks, hypoplastic defects, or surface irregularities were excluded. An acid‑resistant nail varnish (Clarins, Paris, France) was applied to all nonexposed surfaces, leaving a central enamel window of ~ 4 × 4 mm². Specimens were immersed in a demineralizing solution containing 2.2 mM calcium chloride (CaCl₂), 2.2 mM sodium phosphate (NaH₂PO₄), and 50 mM acetic acid [19]. The pH was adjusted to 4.8 using 1 M potassium hydroxide (KOH). The samples were demineralized in the solution at 37°C for 12 hours. This duration and pH were selected to produce initial enamel lesions with a depth limited to less than approximately 100 μm [20].

Then, the specimens were rinsed with deionized water for 10 seconds and air dried. The specimens were then randomized into five groups using a randomization sequence generated in Microsoft Excel. In the current study, the materials were applied using the modified protocol of the previously published study [14] as follows.

- Group 1: Topamine (Dentalife, Melbourne, Australia): ~ 5 µL of the material was applied to the enamel, agitated for 10 seconds.
- Group 2: EXP-SDF (Dentalife, Melbourne, Australia): ~ 5 µL of the material was applied to the enamel, agitated for 10 seconds.
- Group 3: Duraphat (5% NaF varnish; Colgate-Palmolive Co., New York City, NY, USA): A thin layer was applied to the enamel surface using a micro brush for 10 seconds.
- Group 4: ClinproClear (2.1% NaF gel; 3M ESPE Dental Products, St. Paul, MN, USA): A thin layer was applied to the enamel surface using a micro brush for 10 seconds.
- Group 5: Deionized water (DI, negative control): DI was applied to the enamel surface using a micro brush for 10 seconds.

All agents were left on the surface for 60 minutes and then cleaned using water-moistened gauze [14]. The specimens were subsequently subjected to a 14‑day pH-cycling process, following the protocol used in the previous study [19]. The demineralizing solution contained 2.0 mM calcium nitrate [Ca(NO₃)₂], 2.0 mM potassium dihydrogen phosphate [KH₂PO₄], and 75.0 mM acetic acid, adjusted to pH 4.5 using hydrochloric acid. The remineralizing solution comprised 1.5 mM calcium nitrate [Ca(NO₃)₂], 0.9 mM potassium dihydrogen phosphate [KH₂PO₄], 130 mM potassium chloride [KCl], and 20 mM sodium cacodylate [Na(CH_3_)_2_AsO_2_], buffered to pH 7.0. Each cycle involved 6 hours of demineralization followed by 18 hours of remineralization at 37 °C.

Surface microhardness measurements were performed using a Vickers microhardness tester (FM‑800, Future‑Tech Corp., Japan). A 100 g load was applied for 15 seconds [21]. Measurements were obtained at three experimental time points: baseline, after demineralization, and after completion of the pH cycling regimen. Each specimen was divided into four quadrants, with one indentation placed in each quadrant at least 200 µm apart. The mean of the four measurements represented the specimen’s surface microhardness at each time point. The %SMHR was calculated using Equation 1 [22].


$$\mathrm{\%SMHR}=100\times \frac{\mathrm{SM}H_{\mathrm{Final}}-\mathrm{SM}H_{\mathrm{Demin}}}{\mathrm{SM}H_{\mathrm{Initial}}-\mathrm{SM}H_{\mathrm{Demin}}}$$ Equation 1

where SMH_Initial_, SMH_Demin_, and SMH_Final_ represent the Vickers sur-face microhardness values (VHN, Vicker hardness number) measured at baseline, after demineralization, and after completion of the pH-cycling regimen, respectively.

The representative specimen from each group was examined under a stereomicroscope (Leica Zoom 2000, Leica Microsystems, Wetzlar, Germany). Following pH cycling, specimens in the DI control group exhibited extensive surface degradation, rendering the surface microhardness assessment invalid. Therefore, the DI group was excluded from the final analysis of %SMHR [22].

### Raman spectroscopy

Raman spectroscopy was conducted from a representative specimen to evaluate mineral changes in enamel surfaces at baseline, post‑demineralization, and after 14 days of pH cycling. This test was a qualitative assessment (*n* = 1 per group). A Raman imaging microscope (DXR3xi, Thermo Scientific, Waltham, MA, USA) was used to acquire spectra from the central region of three representative specimens per group. Spectra were collected using a laser power of 12.0 mW, an acquisition rate of 325 Hz, 20 cumulative scans, and a 50× objective lens. Imaging was performed over an area of 520 µm with a spatial resolution of 5 µm per pixel. Mineral content was assessed using the phosphate ν₁ peak at 960 cm^–^¹.

### X-ray diffraction analysis

Crystalline phase analysis was performed using X‑ray diffraction (XRD) on the specimen surface. This test was a qualitative assessment (*n* = 1 per group). Measurements were conducted using a Bruker D8 Advance diffractometer (Bruker AXS, Karlsruhe, Germany) with Cu Kα radiation (λ = 1.5418 Å), operated at 40 kV and 40 mA. Diffraction patterns were recorded over a 2θ range of 20–60°, with a step size of 0.02° and a scanning speed of 0.2 seconds per step. Phase identification and verification were performed using the International Centre for Diffraction Data database (ICDD, PDF‑5+ 2026).

### Scanning electron microscopy and energy‑dispersive X‑ray spectroscopy

Surface morphology and elemental composition were examined using scanning electron microscopy (SEM) combined with energy‑dispersive X‑ray spectroscopy (EDX). This test was a qualitative assessment (*n* = 1 per group). The representative specimen was sputter coated with gold using a Q150R coater (Quorum Technologies, UK) at 23 mA for 45 seconds. Imaging was carried out using a field‑emission SEM (JSM‑7800F, JEOL, Japan) equipped with EDX (X-Max 20, Oxford Instruments, Abingdon, UK), operated at 10 kV under high‑vacuum conditions, at magnifications of 10,000× and 30,000×. For each specimen, EDX mapping was performed by averaging the elemental composition (%atom) from three mappings (4 × 4 µm) on the surface.

### MTT test for SDF

The high concentrations of fluoride and silver ions in EXP-SDF may raise concerns regarding potential cytotoxic effects. Therefore, the present study evaluated the cytotoxicity of EXP-SDF compared with Topamine using the MTT assay, following the protocol of the previous studies [23, 24]. Human dental pulp stem cells (hDPSCs) were cultured at a density of 5000 cells/cm^2^ per well in Dulbecco’s Modified Eagle Medium (Gibco, Thermo Fisher Scientific, Waltham, MA, USA), supplemented with 10% fetal bovine serum and 1% penicillin/streptomycin (standard culture medium), at 37°C in a 5% CO_2_ atmosphere. The cells were treated with Topamine and EXP-SDF at concentrations of 1:100, 1:1000, and 1:10,000. These dilution ratios were selected based on a previous study, which reported that SDF concentrations exceeding a 1:1000 dilution may exhibit cytotoxic effects [25]. Cells cultured in standard culture medium without treatment served as the control group.

All cells were incubated at 37°C with 5% CO_2_ for 3 days. The hDPSCs were incubated with 0.2% 3-(4,5-dimethylthiazolyl)-2,5-diphenyltetrazolium bromide (MTT) solution (Sigma-Aldrich, St. Louis, MO, USA) at 37°C for 4 hours. The reaction was terminated using 200 µL of dimethylsulfoxide (Sigma-Aldrich, St. Louis, MO, USA). The color of the resulting product was measured using a microplate reader (Multiskan, Thermo Scientific, Waltham, MA, USA) at 450 nm. Four independent experiments were performed. Cell viability was expressed as a percentage relative to the untreated control group (Equation 2). Medium without cells was used as a blank for background correction.


$$\mathrm{Relative}\;\mathrm{cell}\;\mathrm{Viability}=100x\frac{\mathrm{OD}\;\mathrm{of}\;\mathrm{the}\;\mathrm{test}\;\mathrm{group}}{\mathrm{OD}\;\mathrm{of}\;\mathrm{the}\;\mathrm{control}\;\mathrm{group}}$$ Equation 2

where OD is the optical density.

### Statistical analysis

Numerical results are presented as the mean and 95% confidence interval (CI). Statistical analysis was performed using Prism version 11.0.0 (93) for macOS (GraphPad Software, Boston, MA, USA). Data normality was assessed using the Shapiro–Wilk test. Between-group differences in surface microhardness were analyzed using one-way ANOVA followed by Tukey HSD (Honestly Significant Difference) test. Within-group changes in surface microhardness across time points were analyzed using one-way repeated-measures ANOVA followed by Tukey’s HSD post hoc test. For the MTT assay, two-way ANOVA was used to evaluate the effects of dilution and SDF formulation on cell viability. Statistical significance was set at *p* < 0.05.

## Results

### Materials characterization

The Ag concentrations and pH for Topamine and EXP-SDF were reported as descriptive results. The Ag concentrations were 243,320 ppm and 220,160 ppm for Topamine and EXP-SDF, respectively. Additionally, the pH of Topamine and EXP-SDF was 9.07 ± 0.03 and 7.69 ± 0.07, respectively. For fluoride concentration, EXP-SDF exhibited the highest concentration (138,520 ± 1640 ppmF), which was significantly higher than that of all other groups (*p* < 0.001). Topamine showed a mean concentration of 68,898 ± 462 ppmF, which was significantly higher than that of the NaF groups (*p* < 0.001). Duraphat (20,932 ± 743 ppmF) demonstrated a significantly higher fluoride concentration than ClinproClear (10,574 ± 265.10 ppmF) (*p* < 0.001).

### %SMHR of enamel

The Vickers surface microhardness of enamel in all groups was comparable at baseline and after demineralization (*p* > 0.05; Table 2). After 2 weeks of pH cycling, a significant increase in surface microhardness was observed in the Topamine and EXP-SDF groups (*p* < 0.01). However, ClinproClear showed a significant decrease in surface microhardness (*p* < 0.01), while Duraphat showed no significant change in surface microhardness (*p* = 0.0642). In the control group (DI), a breakdown of the enamel surface was observed. Hence, the surface microhardness after pH cycling in the DI group could not be measured precisely, and the %SMHR could not be calculated.

**Table 2:** Surface microhardness (VHN) at baseline (initial), after demineralization, and after 2 weeks of pH cycling.

Time/materials	Topamine	EXP-SDF	Duraphat	ClinproClear	DI
Initial	344.3(10.15) A,a	348.5 (7.8) A,a	345.4 (12.1) A,a	348.4 (10.3) A,a	349.8 (10.3) A,a
After demineralization	138.6 (8.4) A,b	137.3 (11.9) A,b	130.3 (8.5) A,b	132.6 (4.3) A,b	134.8 (5.8) A,b
After pH cycling for 2 weeks	212.1 (9.8) A,c	209.9 (12.0) A,c	115.1 (10.8) B,b	36.4 (4.5) C,c	NA

Identical uppercase letters within the same row indicate no significant difference among materials at the same time point (*p* > 0.05). Identical lowercase letters within the same column indicate no significant difference within the same material across time points (*p* > 0.05). After pH cycling, the DI group exhibited severe surface breakdown, so the microhardness value could not be recorded (NA). EXP: experimental; SDF: silver diamine fluoride; DI: deionized water.

**Figure 2:**
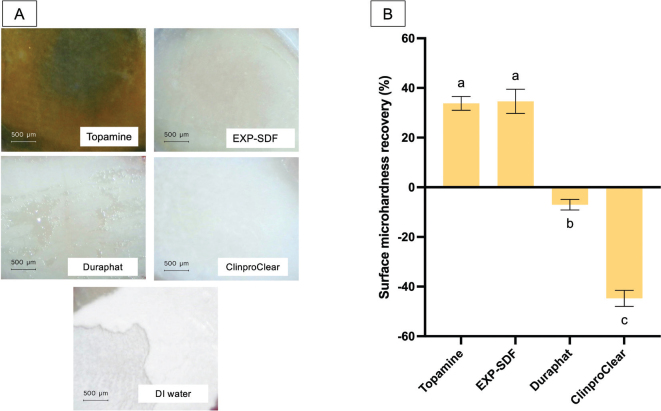
(A) The surface of representative specimens from each group after pH cycling. (B) Surface microhardness recovery for each group. Different letters indicate a significant difference (p < 0.05). The error bars are 95% CI (n = 9).

The highest %SMHR was observed in the Topamine (33.8 ± 3.6%) and EXP-SDF (34.6 ± 6.3%) groups (Figure 2). No significant difference between the %SMHR of Topamine and EXP-SDF was detected (*p* = 0.9816). In contrast, Duraphat and ClinproClear exhibited lower and negative %SMHR values (−7.0 ± 2.7% and −44.8 ± 4.2%, respectively). The %SMHR of Topamine and EXP-SDF was significantly higher than that of Duraphat and ClinproClear (*p* < 0.05).

Representative enamel specimens showed distinct differences in surface appearance after treatment (Figure 2). The specimen treated with Topamine exhibited clear black staining, whereas the specimen treated with EXP-SDF showed slight yellowish discoloration. In contrast, Duraphat and ClinproClear produced minimal color change. Surface breakdown was clearly observed in the DI group.

### Raman spectroscopy

**Figure 3:**
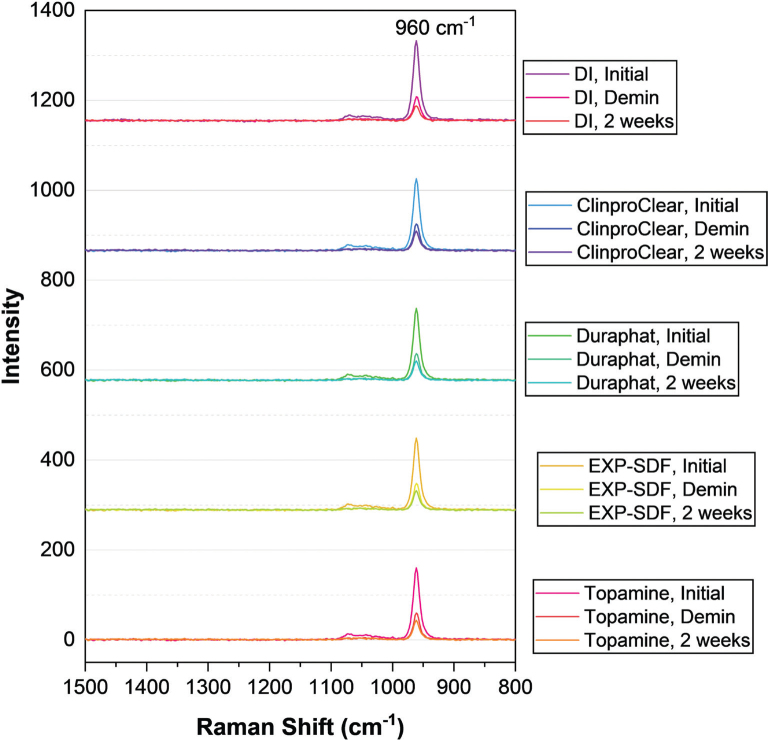
Raman spectra of the representative material obtained at baseline (initial), after demineralization, and after treatment followed by pH cycling for 2 weeks.

Raman spectroscopy showed mineral loss following demineralization, with similar mineral recovery levels across treatment groups after pH cycling (Figure 3). All groups exhibited a prominent phosphate ν₁ PO₄³ ^–^ peak at 960 cm^–^ ¹ at baseline. Peak intensity decreased markedly after demineralization but remained at comparable levels after pH cycling across all groups.

### XRD analysis

**Figure 4:**
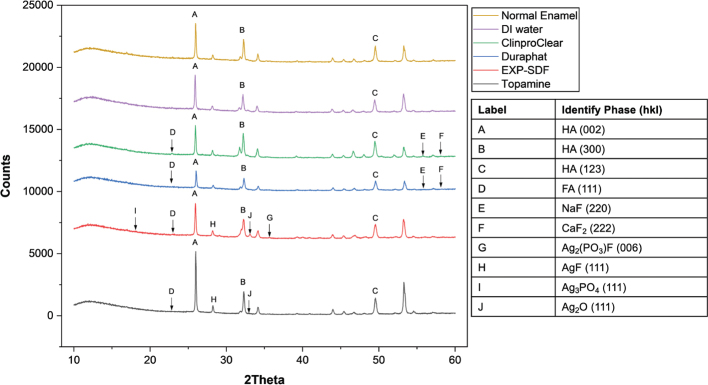
X-ray diffraction (XRD) patterns of enamel specimens from each group after 2 weeks of pH cycling. Major diffraction peaks are indexed to their corresponding Bragg reflections (hkl; three-digit Miller indices).

The XRD spectra of representative enamel specimens after pH cycling in each group are shown in Figure 4. Hydroxyapatite or HA (Ca_10_(PO_4_)_6_(OH)_2_) diffraction peaks were observed at 25.879°, 32.939°, and 49.490° across all groups, consistent with (002), (300), and (123) Bragg reflections. The specimen in the DI group showed only HA peaks. Specimens treated with Topamine, EXP-SDF, Duraphat, and ClinproClear showed a fluorapatite (Ca_5_(PO_4_)_3_F) peak at 22.874°, corresponding to the (111) Bragg reflection. The specimens treated with Duraphat and ClinproClear exhibited a NaF peak at 56.40°, consistent with the (220) Bragg reflection of the cubic structure of NaF. Similarly, a calcium fluoride (CaF_2_) peak was observed in Groups Duraphat and ClinproClear.

In both Topamine and EXP-SDF, which contain silver, the peak at 28.363° coincided with the (111) Bragg reflections of silver fluoride (AgF), and the peak at 32.90° coincided with the (111) Bragg reflections of silver oxide (Ag_2_O). However, the peaks at 36.434° for silver phosphorus oxide fluoride (Ag_2_(PO_3_)F) and 20.884° for silver phosphate (Ag_3_PO_4_) were observed only in the specimen from the EXP-SDF group.

### SEM and EDX

**Figure 5:**
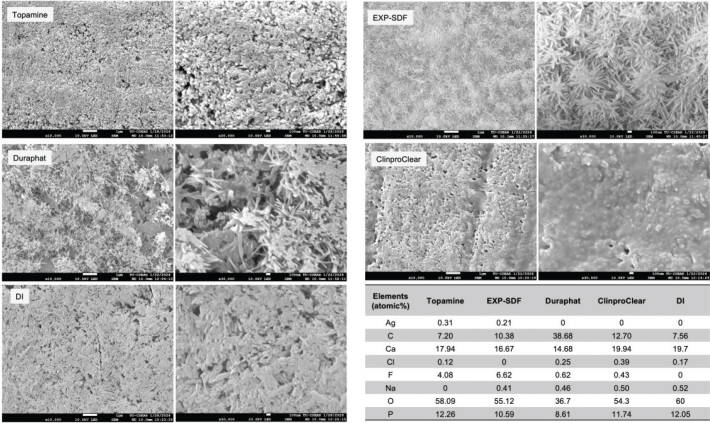
Representative SEM images of enamel specimens after 2 weeks of pH cycling. Elemental composition was averaged from EDX maps of three surface areas. SEM: scanning electron microscopy; EDX: energy-dispersive X-ray spectroscopy.

SEM images of the representative specimen from the DI group exhibited disorganized enamel mineral crystals (Figure 5). In contrast, enamel treated with Topamine exhibited dense mineral precipitation and a relatively smooth surface with minimal structural disruption. The EXP-SDF group showed sharp, needle-like crystalline precipitates distributed across the enamel surface. The Duraphat group also exhibited mineral precipitation, but it was less dense than that observed in the Topamine and EXP-SDF groups. In the ClinproClear group, a porous and pitted surface morphology was observed. EDX analysis detected silver only in the Topamine and EXP-SDF groups. In addition, fluoride levels on enamel treated with Topamine and EXP-SDF were higher than those in the Duraphat and ClinproClear groups. Neither Ag nor F was detected in the DI group.

### MTT test for SDF

**Figure 6:**
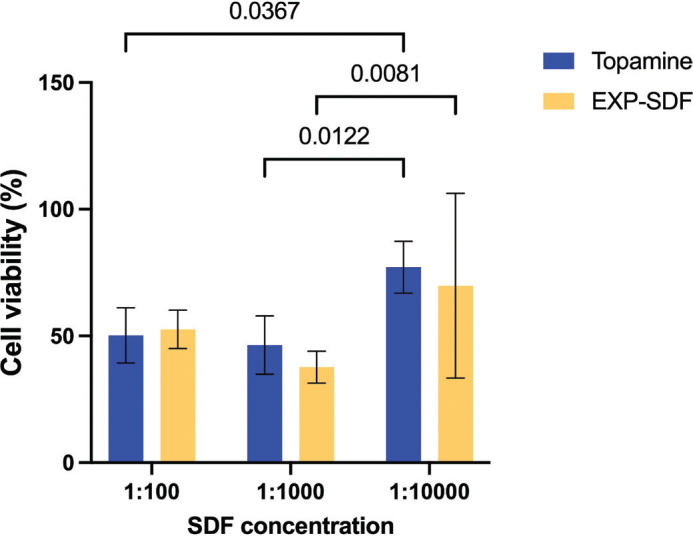
Cell viability of hDPSCs treated with different concentrations of Topamine and EXP-SDF. Error bars are 95% CI (n = 4). Lines indicate p < 0.05. EXP: experimental; SDF: silver diamine fluoride; hDPSCs: human dental pulp stem cells.

The highest cell viability was observed at a concentration of 1:10,000 for Topamine (77 ± 6%) and EXP-SDF (70 ± 20%) (Figure 6). For Topamine, the viability at a concentration of 1:10,000 was significantly higher than that at 1:100 (50 ± 7%) (*p* = 0.0367) and 1:1000 (46 ± 7%) (*p =* 0.0122). For EXP-SDF, the cell viability at a 1:10,000 concentration was also higher than that of the lower concentration at 1:1000 (38 ± 3%) (*p* = 0.0367). Two-way ANOVA demonstrated that the type of SDF formulation exhibited no effect on cell viability (*p* = 0.3189), while the effect of concentration was significant (*p* < 0.01).

## Discussion

The present study compared the *in vitro* enamel remineralization of the experimental SDF formulation (EXP-SDF) with a commercial 38% SDF (Topamine) and topical NaF products (Duraphat and ClinproClear). EXP-SDF exhibited a %SMHR comparable to Topamine but significantly higher than those of Duraphat and ClinproClear. The *in vitro* cytotoxic effect of Topamine was comparable to that of EXP-SDF. Hence, the null hypothesis was partially rejected.

The silver and fluoride concentrations, as well as the pH of EXP-SDF, fell within the ranges reported for commercially available SDF products [26]. The fluoride concentration and pH of Topamine in the present study were also within the range of those reported in the previous study (approximately 58,000–90,000 ppm fluoride and pH 7.9–8.1) [26]. Variations in concentration and pH may be attributable to storage conditions or water evaporation, leading to increased ion concentration over time. Hence, shelf-life assessment is needed for EXP-SDF in future studies to confirm chemical stability.

The present study suggested that SDF promoted significant remineralizing effects on demineralized enamel, which is in agreement with the previous systematic review [8]. Despite differences in the initial fluoride concentrations of EXP-SDF and Topamine, both materials showed no detectable differences in remineralization outcomes in the current study. It was hypothesized that the high fluoride content of both SDF formulations may promote dense mineral precipitation on the surface [27]. A limitation of the microhardness test is that the result may reflect mineralization limited to the outermost layer of enamel [28]. Future studies should employ micro-CT or transverse microradiography (TMR) to assess differences in lesion depth and quantify mineral density within the treated enamel.

The higher %SMHR observed in the SDF groups compared with Duraphat and ClinproClear may be due to the high fluoride availability and the presence of silver ions [7, 14, 29]. Silver ions may interact with phosphate and chloride ions in the oral environment, forming less soluble compounds such as silver phosphate (Ag₃PO₄) and silver chloride (AgCl) [7, 30]. These silver precipitates may subsequently accumulate on demineralized enamel, forming a mineral reservoir [31, 32]. Additionally, silver phosphate may provide a local source of phosphate that supports sustained surface‑level remineralization under repeated acid challenge [7, 14, 32, 33].

The enamel surface treated with EXP-SDF and Topamine showed less widening of the interprismatic/intercrystalline spaces and denser mineral precipitation than that of Duraphat and ClinproClear. EDX findings further suggested that the surface precipitates in the EXP-SDF and Topamine groups contained Ag- and F-containing mineral phases. These findings are consistent with a previous study [32] demonstrating that SDF promotes the formation of fluorapatite and metallic silver, among other silver and fluoride-containing mineral phases that increase resistance to acid attack. These precipitates may help preserve enamel structure, thereby resulting in greater measured %SMHR compared with NaF products. Additionally, the SEM result demonstrated multiple elongated or rod-shaped crystals on representative enamel treated with EXP-SDF. The characteristics may resemble those of fluorohydroxyapatite crystals reported in previous studies [34–36].

The formation of silver salts after applying SDF was confirmed by XRD, consistent with the previous study [37]. The present study, however, fails to detect the AgCl peak, in contrast to previous studies [14, 35, 37]. This discrepancy could be attributed to the variation in the composition of the remineralizing solution in the pH cycling protocol. The precipitation of AgCl was typically detected in the pH cycling model using a remineralizing solution with a high chloride concentration, such as Tris-buffered saline or human or artificial saliva [37]. The XRD result in the present study also detected silver phosphorus oxide fluoride [Ag₂(PO₃)F] in the enamel treated with EXP-SDF. The pH and fluoride concentration in EXP-SDF may favor the precipitation of this compound. Further study is, however, needed to clarify its origin and mechanisms.

For NaF products, ClinproClear exhibited inferior *in vitro* enamel remineralization to Duraphat. This may be partly attributable to its lower fluoride concentration relative to other materials, which could reduce fluoride availability and remineralization potential [38, 39]. Additionally, the increased contact time of NaF varnish on the demineralized enamel may be associated with increased remineralizing effects [40]. Hence, the absence of natural resin in ClinproClear may reduce retention time and contact time on the enamel surface, thereby limiting its protective effect during cycling. However, these findings should be interpreted in the context of the *in vitro* study model, which may not fully reflect the clinical situation [41].

Raman spectroscopy failed to demonstrate an increase in HA peaks after pH cycling in experimental groups, particularly in the SDF groups. The possible explanation is that silver-containing pigmented reaction products attenuate the Raman signal, thereby reducing peak detectability [33]. In addition, Raman spectroscopy primarily reflects near-surface changes (approximately 2–25 µm) [42]. It was, however, reported that SDF-associated mineral deposition contributing to microhardness recovery may extend to ~20 µm in sound enamel and exceed 600 µm in porous lesions [42]. A limitation of this study is that the origin of individual specimens was not tracked. Therefore, a potential clustering effect may occur when specimens are obtained from the same tooth. Future studies should account for specimen origin to ensure statistical independence among observations.

The *in vitro* toxicity of EXP-SDF and Topamine showed a similar concentration‑dependent pattern. The present study indicated that the 1:10,000 dilution of both EXP-SDF and Topamine showed higher cell viability than the lower dilution ratio. The result also agrees with a previous study, which indicated that SDF exhibited minimal *in vitro* toxicity toward hDPSCs at dilutions of 10^–^ ⁴ and higher [25]. Although EXP-SDF contains a higher concentration of fluoride, it showed slightly lower cell viability than Topamine. This may be because the test was conducted at relatively low SDF concentrations. However, the result should be interpreted with caution, as the metallic color from SDF reactions may affect the optical density readings [43].

Although the World Health Organization (WHO) has included 38% SDF in its Model List of Essential Medicines to promote global accessibility and affordability, SDF is not yet registered or commercially available in all countries [44]. Developing more cost-effective manufacturing methods may help overcome economic and regulatory barriers that limit its wider adoption. Lower production costs could offset additional regulatory expenses, thereby improving the cost-effectiveness of community-based caries prevention programs [9]. In addition, reduced material costs may enhance the financial sustainability of SDF use in clinical practice, particularly in settings with reimbursement barriers [45]. The reduced metallic discoloration observed with the experimental formulation may further increase acceptance among patients, parents, and caregivers and expand its use in areas with esthetic concerns [10, 11].

Although conventional 38% SDF is effective in promoting enamel remineralization, its major clinical limitation is the black discoloration of treated lesions [44]. The representative specimen treated with EXP-SDF may exhibit less discoloration than the specimen treated with Topamine. This could be due to the different phases of silver salts formed on the specimen [7, 46]. Several alternative formulations and applications to minimize this esthetic drawback were also proposed. Approaches to reduce SDF-induced discoloration include applying potassium iodide to precipitate free silver ions as the yellowish-white silver iodide, thereby reducing the typical black discoloration. However, the use of KI may result in poor long-term color stability and reduced anti-caries efficacy [47, 48]. Another method was to use silver nanoparticles, such as nano silver fluoride, which offer comparable antibacterial and remineralization efficacy with reduced staining [49, 50]. In addition, ammonia-free, water-based silver fluoride formulations have been developed to address limitations of conventional SDF [51]. By replacing ammonia with water, these formulations achieve a near-neutral pH closer to the physiological range, thereby reducing gingival irritation, eliminating unpleasant odor, and potentially improving esthetic outcomes [51, 52]. Future studies should include spectrophotometric measurements to quantify color parameters among different products or approaches that aim to reduce discoloration [53].

Limitations of the present study should be acknowledged. The *in vitro* pH‑cycling model provides well‑controlled conditions but cannot account for other key factors, such as the oral microbiome, biofilm activity, salivary effects, or host responses [41, 54, 55]. Remineralization was assessed primarily using surface microhardness and surface‑oriented analyses, which may not directly measure mineral content or fully analyze the subsurface lesion changes [56]. In addition, although XRD and EDX findings suggested mineral precipitation, precise chemical differentiation remains challenging due to overlapping structural features and analytical detection limits [7, 31, 57].

## Conclusion

Within the limitations of this in vitro study, EXP-SDF demonstrated enamel remineralization efficacy comparable to that of a commercially available SDF product without increasing cytotoxicity. In addition, EXP-SDF exhibited superior remineralization performance compared with professionally applied sodium fluoride products, including 5% NaF varnish and 2.1% NaF gel. These findings support the continued development of EXP-SDF as a promising noninvasive caries-arresting agent and provide a foundation for future *in vivo* and clinical investigations.

## Data Availability

Raw data are available upon request to the corresponding author.
